# Evaluating Clinical Course and Risk Factors of Infection and Demographic Characteristics of Pregnant Women with COVID-19 in Hamadan Province, West of Iran

**DOI:** 10.34172/jrhs.2020.22

**Published:** 2020-08-17

**Authors:** Mahtab Sattari, Saeed Bashirian, Seyedeh Zahra Masoumi, Arezoo Shayan, Ensiyeh Jenabi, Samereh Ghelichkhani, Azam Ali Shirzadeh, Ebrahim Jalili, Shohreh Alimohammadi

**Affiliations:** ^1^Department of Midwifery, School of Nursing and Midwifery, Hamadan university of Medical Sciences, Hamadan, Iran; ^2^Social Determinants of Health Research Center, Hamadan University of Medical Sciences, Hamadan, Iran; ^3^Department of Public Health, School of Health, Hamadan University of Medical Sciences, Hamadan, Iran; ^4^Department of Midwifery, Mother and Child Care Research Center, School of Nursing and Midwifery, Hamadan University of Medical Sciences, Hamadan, Iran; ^5^Autism Spectrum Disorders Research Center, Hamadan University of Medical Sciences, Hamadan, Iran; ^6^Department of Emergency Medicine, School of Medicine Besat Hospital, Hamadan University of medical sciences, Hamadan, Iran.; ^7^Department of Gynecology, School of Medicine, Hamadan University of Medical Sciences, Hamadan, Iran.

**Keywords:** COVID-19, Demography, Risk Factors, Pregnant Women, Coronavirus

## Abstract

**Background:** COVID-19 is a new viral disease with a rapid outbreak. Pregnant women are at a higher risk of contracting viral infections including COVID-19. We aimed to evaluate the clinical course and risk factors of pregnant women diagnosed with COVID 19 in Hamadan Province, west of Iran.

**Study design:** A retrospective cohort study.

**Methods:** The convenience sampling was performed using 50 papers and electronic files of pregnant women diagnosed with COVID-19 according to the WHO’s temporary guidelines. They were hospitalized in health centers and clinics of Hamadan Province. The data-collecting tool employed was a researcher-made questionnaire. The data were analyzed via SPSS software version 19.

**Results:** The mean age of pregnant women with COVID 19 was estimated to be 29.20 ± 5.8 yr and their average gestational age estimated to be 28.8 ± 8.20 weeks. About 32% of them had an underlying disease, 32% a history of influenza, and 40% recently traveled to infected areas. The most common findings were CT scans and multiple mottling and ground-glass opacity chest radiology. The most common symptoms were fever, cough, and shortness of breath. About 8% of the women required ICU hospitalization and the average length of hospital stay was 4.04 ± 2.38 and 29% had premature births. Moreover, 28% of infected mothers had a normal delivery and 20% had a cesarean section.

**Conclusion:** Early diagnosis of Covid-19 disease is essential in pregnant women. Because there is a possibility of worsening complications in the mother and fetus.

## Introduction

Coronavirus 2019 (COVID-19) is a relatively new disease with a high outbreak rate. The virus causes severe pneumonia such that WHO has recognized it as a global public health emergency^1,2^. Coronavirus is a coated single-stranded ribonucleic acid called solar corona due to the presence of 9-12-nm-long spikes on its surface ^3^. There are four main structural proteins on the coating encrypted by the virus genome. One of these proteins is spike (S), which binds to the angiotensin enzyme converter enzyme 2 (ACE2) receptor and mediates further fusion between the host cell membrane and the virus’ coating, leading to virus entry into the host cell^4,5^. Coronavirus can cause a variety of illnesses observed in the forms of a mild cold to severe respiratory illness and even death ^6^. At present, primary epidemiological risk factors for COVID-19 include traveling to the infected and high-risk areas or close contact with the infected individuals within 14 days from the onset of symptoms^1,7^. Common symptoms include fever, cough, myalgia, headache, and diarrhea. Abnormal tests also include chest radiographs, lymphopenia, leukopenia, and thrombocytopenia. Preliminary reports of acute respiratory distress syndrome (ARDS) have also been observed in 17-29% of hospitalized patients^1^.

The virus spreads via infected respiratory droplets, usually through individual to individual(s) or through the contact of mucus with infected surfaces. This virus can lead to pneumonia, ARDS, kidney, and several other organ failures. Elderlies with chronic and severe illnesses such as asthma, diabetes, heart failure, and immune system disorders are more prone to be infected with COVID-19 ^7^. Besides, due to immunophysiological changes during pregnancy, pregnant women are at a higher risk for contracting viral infections, including COVID-19^8,9^. Consequently, the pathogenesis of COVID-19 infection may be similar to SARS-CoV-1 and the risk of vertical COVID-19 transmission may be as high as SARS-CoV-1 ^6^.

SARS infection during pregnancy has adverse maternal and neonatal complications associated with adverse complications including spontaneous abortion, preterm delivery, intrauterine growth restriction, tracheal intubation, intensive care unit hospitalization, renal failure, and intravascular coagulation ^1,10^. Nevertheless, the complications and deaths resulting from COVID-19 in pregnant women and their infants are less compared to those of other viruses in the Corona family such as SARS and MERS^11,12^. Two reports of 18 pregnant women with COVID-19 respectively confirmed that they were all infected in the third trimester and had clinical findings similar to those of non-pregnant infected adults. There were also cases of fetal distress and preterm delivery ^1^. Symptomatic care along with an early diagnosis of the disease in pregnant mothers, a strong immune system, and general well-being can be effective determinants in improving maternal and fetal prognosis ^8^. There is no conclusive evidence of complications, symptoms, and risk factors for pregnant mothers, especially in Iran, which is one of the countries with the highest prevalence of the disease.

We aimed to evaluate the clinical course and risk factors of pregnant women diagnosed with COVID 19 in Hamadan Province, west of Iran.

## Methods

The present study was conducted as a retrospective cohort on pregnant mothers hospitalized diagnosed with COVID-19 in hospitals of Hamadan Province, Iran, in 2020. Sampling of all pregnant women suspected of having coronavirus disease was performed in the Obstetrics and Gynecology Department from January 6, 2020 to June 21, 2020. The name lists of all pregnant mothers who were diagnosed with a temporary WHO guideline for COVID-19 were provided via the infection control center, in coordination with the departments and security office of each hospital.

The convenience sampling was carried out among 50 cases of hospitalized pregnant women diagnosed with COVID-19. To gather the needed information, the electronic files along with the paper copies of the files in each hospital’s archive were scanned. A few professionals familiar with midwifery and clinical cases of COVID-19 visited the hospitals several times to observed and maintain health protocols and prevention orders. These professionals carefully examined the files, extracted the necessary information, and registered them. Patients’ files were examined for demographic information, symptoms, and clinical course of the disease and risk factors. To this end, these mothers’ information was examined through their electronic files and paper copies of the files. The collection of epidemiological, demographic, clinical, laboratory, therapeutic, and results from medical records data were extracted using a standard data set. The results of the chest radiology and PCR tests were also examined.

Fever, secondary infection, pneumonia, acute kidney damage, and ARDS were assessed based on the following criteria. The minimum fever was defined as an armpit temperature of at least 37.3 ℃. Sepsis and septic shock are examined according to the Definition for Sepsis and Septic Shock the 2016 Third International Consensus ^13^. Secondary infection was assessed based on patients with clinical signs or post-hospitalization blood sample ^14^. Acute renal impairment was also assessed based on KDIGO clinical practice guidelines^15^. Acute myocardial infarction was diagnosed if the serum level of cardiac biomarkers (e.g., high-sensitivity cardiac troponin I) was higher than 99% or new evidence was available on the electrocardiography ^13^. Finally, ARDS was assessed based on the Berlin Definition ^16^.

Data were analyzed using SPSS software version 19 (Chicago, IL, USA) and descriptive statistics including mean and standard deviation and relative frequency and percentage in the form of frequency tables.

## Results

The present study was conducted to evaluate 50 hospitalized pregnant women with an age range of 18-38 yr diagnosed with COVID-19. Besides, their average gestational age was 28.42 ± 8.20 yr. Among these women, 24 mothers gave birth, 18 of whom were multi-pars, 55.6 had a history of cesarean section, and 44.4 had a history of normal vaginal delivery. Moreover, 16 of them had a background of underlying diseases and influenza and 40% had an account of recent travel to high-risk and infected areas. The most-reported findings in CT scans were patients with multiple mottling and ground-glass opacity. Other demographic characteristics of the infected pregnant mother, the severity of the disease, the type of the underlying disease, and the chest radiographic findings are shown in Table 1.

**Table 1:** Demographic characteristics and clinical results of pregnant women with COVID-19 (n = 50)

**Categorical variables**	**Number**	**Percent**
History of smoking	3	6.0
Previous delivery method		
Vaginal	8	44.4
Caesarean section	10	55.6
Background disease	16	32.0
The type of background disease		
Diabetes	1	6.2
Hypertension	2	12.5
Heart disease	2	12.5
Other diseases	11	68.8
History of influenza	16	32.0
Recent travel to high-risk areas	20	40.0
Clinical classification of COVID- 19 in patients		
Mild	24	48.0
Medium	18	36.0
Severe	8	16.0
CT-scan finding		
Consolidation	13	26.0
Infiltration	14	28.0
Bilateral pneumonia	13	26.0
Multiple mottling and ground-glass opacity	15	30.0
**Continuous variables**	**Mean**	**SD**
The age range (18-38)	29.20	5.80
Gestational age (week)	28.42	8.20
Number of live births	1.41	1.09
Number of abortions	0.20	0.49

Examination of the signs and symptoms of pregnant women with COVID-19 upon admission to the hospital showed that the average oxygen saturation percentage was 91.62 ± 7.58 and their most common symptoms at the time of admission were fever (60.0%). Coughing and Dyspnea were 54% each. Pneumonia was also observed in 38% of pregnant women at the time of admission to the hospital (Table 2). Laboratory findings showed an increase in white blood cell counts, C-reactive protein, and alanine aminotransferase, and a decrease in platelet, hemoglobin, and red blood cell counts (Table 3).

**Table 2:** Signs and symptoms of pregnant women with COVID-19 when admitted to the hospital (n = 50)

**Variables**	**Number**	**Percent**
Sepsis	1	2.0
Pneumonia	19	38.0
Acute respiratory syndrome	2	4.0
Acute damage to the heart	2	4.0
Coagulopathy	3	6.0
Hypoproteinemia	2	4.0
Acidosis	4	8.0
Fever	30	60.0
Cough	27	54.0
Muscular pain	9	18.0
Fatigue	6	12.0
Diarrhea	2	4.0
Nausea	7	14.0
Vomit	2	4.0
Dyspnea	27	54.0
Tachycardia	6	12.0
O_2_ Saturation percent	**Mean**	**SD**
	91.62	7.58

**Table 3:** Laboratory findings in pregnant women with COVID-19 (n = 50)

**Laboratory indicators**	**Normal range**	**Mean**	**SD**	**Increased number**		**Reduced number**	
				**Number**	**Percent**	**Number**	**Percent**
Platelet count (10^3^/UL)	150 to 450	217	87.06	-	-	12	24.0
Red blood cells (10^6^/UL)	3.0 to 5.0	4.10	0.42	-	-	7	14.0
White blood cells (10^9^/Ul)	5 to 13	10.66	4.64	22	44.0	-	-
Lymphocyte count (%)	20 to 40	19.58	8.57	-	-	5	10.0
Hemoglobin (g/dl)	10 to 15	11.90	1.86	-	-	15	30.0
C-Reactive Protein (mg/dl)	0 to 2	1.84	0.99	14	28.0	-	-
Creatinine (mg/dl)	0.4 to 0.9	0.77	0.18	-	-	-	-
Alanine aminotransferase (U/L)	2 to 25	23.49	18.02	5	10.0	-	-

Furthermore, regarding the effect of COVID-19 on the complications of pregnancy in infected women, the results showed that infected pregnant women were hospitalized for an average of 4.04 ± 2.38 days and 8% of them required hospitalization in the ICU. There were two maternal deaths due to COVID-19. Of the studied population, 24 infected mothers gave birth during this period, 25 babies were born, and seven cases of COVID 19 were reported among the newborns according to Covid-19. (Table 4). Details of pregnancy results and the birth rate of neonatal Apgar score are shown in Table 4. The most commonly used drugs to treat pregnant women with COVID-19 were hydroxychloroquine, oseltamivir, Kaletra, azithromycin, and vancomycin.

**Table 4:** Pregnancy results in mothers with COVID-19 (n=50)

**Categorical variables**	**Number**	**Percent**
Number of hospitalized patients in the ICU	4	8.0
Mother's death	2	4.0
Vaginal delivery	14	28.0
Caesarean section	10	20.0
They have not given birth yet	26	52.0
Preterm delivery	7	29.2
Infection of the newborn according to Covid-19	7	28.0
**Continuous variables**	**Mean**	**SD**
Number of hospitalizations	4.04	2.38
Apgar score (first minute)	8.12	1.61
Apgar score (fifth minute)	9.24	1.20

## Discussion

COVID-19 has spread rapidly in most parts of the world, infecting and even killing many people. Pregnancy is a physiological condition that exposes women to viral infections^17^. Beyond the impact of COVID-19 infection on pregnant women, there are concerns about the potential impact on the fetus and the infant. Accordingly, the pregnant women should be examined more closely due to physiological and immunological changes during pregnancy, childbirth, the presence of the fetus, and even the possibility of the fetus or infant infection. In this regard, all pregnant women diagnosed with COVID-19 in Hamadan Province referred to hospitals or were hospitalized during different periods of pregnancy with different gestational ages were examined. Mothers were in the different stages of pregnancy, ranging from early first trimester to the late third trimester. Several mothers referred only for the delivery. According to the patients’ files, clinical, and laboratory evidence, the women were in the suspected and infected category, receiving medical treatment, care, and support. The average age of pregnant women admitted to hospitals was 29.20 ± 5.80 yr and their gestational age average was approximately 28 weeks. Most of the infected pregnant women were young; most of them had experienced previous pregnancy; and 55% had a history of cesarean delivery.

In China, Chen et al. investigated nine pregnant women with COVID-19 pneumonia between the ages of 26 and 34 years. Their age range was similar to that of pregnant women in this study ^18^. Studies on the history of the disease showed that among women with COVID-19, there was a history of underlying diseases such as diabetes, hypertension, and cardiovascular disease and about one-third of the women surveyed had a history of the influenza disease. Other studies have looked less strictly at maternal morbidity, which may be due to younger mothers having a lower risk of underlying diseases. Given that the initial prevalence of COVID-19 had increased dramatically in some Iranian cities, about 40% of the infected women had recently traveled to these areas. Therefore, it can be suggested that traveling to the high-risk areas pose them to a higher risk of infection. Wang et al. presented a case report from a 28-yr-old pregnant mother diagnosed with COVID-19 who had an ongoing 30-weeks pregnancy. They showed that the mother had recently traveled to the infected areas ^8^. According to clinical evidence, examinations, and tests, most cases of the disease, or nearly half of all pregnant mothers, showed only mild symptoms, with only 16% experiencing severe illness symptoms. In other studies, most women did not have severe symptoms, and only one pregnant woman within the Zhang study had severe disease, and the rest were asymptomatic ^18-20^.

Sixteen percent of pregnant women had no symptoms at all ^12^. In many cases, chest radiography and CT scans were carried out to confirm the diagnosis of COVID-19. In this regard, most of the cases were reported with multiple mottling and ground-glass opacity. The infiltration method is the second most frequently used method in this regard. In line with these results, other studies have regularly reported these two findings in the CT scan and examination of the lungs of infected pregnant women while hospitalized^8,17,20,21^. Pregnant women infected with COVID-19 were suffering from a wide range of symptoms upon admission to the hospital. These symptoms included fever, cough, muscle aches, fatigue, diarrhea, nausea, vomiting, and dyspnea, tachycardia, pneumonia, and ARDS. A small number of patients also had hypoproteinemia, coagulopathy, and acute heart damage. Among them, fever, cough, shortness of breath, and pneumonia were the most common symptoms of COVID-19 infection in pregnant women, respectively. In most studies, fever, cough, and shortness of breath have been reported in pregnant women ^18-20,22^. Moreover, the symptoms of pregnant women infected with the COVID-19 virus do not seem to be different from those infected of other backgrounds and non-pregnant adults. However, other studies did not report severe pneumonia in pregnant women^18,19^.

The Yang et al study also found no evidence of greater susceptibility to COVID-19 infection in pregnant women and that people with COVID-19 infection were more likely to develop severe pneumonia ^17^. Oxygen saturation percentage in these women during admission and hospitalization was 91.62 ± 7.58, although these values ​​increased or decreased in different patients during regular inspections. In patients with increased severity of symptoms and worsening of the disease, the oxygen saturation percentage declined. Unfortunately, among postpartum women, there were two cases of maternal death from COVID-19, with an acute decrease in oxygen saturation in the later stages of the disease. The pregnant mother admitted to the emergency had an oxygen saturation percentage above 90% (i.e., 97%) ^8^. Despite two cases of maternal death in our study, in most studies, not maternal mortality from COVID 19 was reported^8,12,18,19,23^ and only one case of maternal and neonatal death was reported ^22^. The rank of COVID-19 is 13th among 20 major causes of death. Unfortunately, the rank of COVID-19 is 13th among 20 major causes of death ^24^. Examining laboratory indicators of patients also revealed an increase in white blood cells, thrombocytopenia, lymphopenia, C-reactive protein, and alanine aminotransferase (U / L). Laboratory data from the Chen and YANG study also showed that most pregnant women diagnosed with COVID-19 had lymphopenia and increased C-response protein^17,18^. The infected women were hospitalized for only about 4 days and required clinical care and just few needed ICU hospitalization. Similarly, the most common drugs used to treat pregnant mothers with COVID-19 infection were hydroxychloroquine, oseltamivir, Kaletra, azithromycin, and vancomycin^8,12^.

Of all the referred women, 24 were transferred to medical centers for delivery, of which 10 required cesarean delivery and the rest went into labor naturally. The results showed that about 29% of women gave birth prematurely. In addition to our results, several studies, have noted an increase in preterm labor, which should be considered as a significant issue^12,22,23^. Apparently, in most studies, due to instability of the maternal and fetal status, cesarean delivery is preferred^12,18,19,22^. Although timely termination of pregnancy in pregnant women with COVID-19 does not increase the risk of preterm delivery and neonatal asphyxia, it is beneficial for the treatment and rehabilitation of the mother ^19^. In studies on infants ^18-21^, the results determined that the average score of Apgar in the first and fifth minutes was optimal; however, the score of Apgar in the fifth minute improved compared to that of the first minute. In the present study, the results of the neonatal PCR test showed that seven infants were infected with the coronavirus postpartum and thus were monitored in the NICU. Nevertheless, the general condition of all infants was satisfactory. In most studies, the condition of newborns and their postpartum Apgar score was reported to be optimal in preterm deliveries of infected mothers^8,18-21,23^. Contrary to our results, there is no evidence of breast milk infection. Transmission of the disease through breast milk has still been studied in another study ^22^.

## Conclusion

Apparently, the disease will worsen and even lead to death in mothers diagnosed with COVID-19, as well as infant infection. Furthermore, despite the similarity of the symptoms of pregnant women with other non-pregnant women, it seems that the possibility of premature delivery, cesarean delivery, fetal distress, and the effect on the Apgar score of newborns is not far-fetched. Consequently, understanding the symptoms, clinical course, and paraclinical findings in these patients can be practical in diagnosis, treatment, and medical measures to improve prognosis in mothers, fetuses, and infants.

## Acknowledgements

The authors are grateful to the support of Hamadan University of Medical Sciences, Hamadan Health Center, the participants of the study, and all those who contributed to this research.

## Conflict of interest

No potential conflict of interest was reported by the authors

## Funding

This study was funded by the Hamadan University of medical sciences, (ID: 990119154).

## Highlights

- Preterm delivery, cesarean section and fetal distress are possible complications of pregnancy with COVID-19.
- The most common symptoms in pregnant women with COVID-19 were fever, cough, and respiratory distress.
- Average oxygen saturation percentage was 91.62 ±7.58 in pregnant women with COVID-19.
- Infected infants were also observed among infants born to women with COVID-19.
